# Experimental Characterization of the Thermal Conductivity and Microstructure of Opacifier-Fiber-Aerogel Composite

**DOI:** 10.3390/molecules23092198

**Published:** 2018-08-30

**Authors:** Hu Zhang, Chao Zhang, Wentao Ji, Xian Wang, Yueming Li, Wenquan Tao

**Affiliations:** 1 Key Laboratory for Strength and Vibration of Mechanical Structures, Shaanxi Key Laboratory of Environment and Control for Flight Vehicle, School of Aerospace, Xi’an Jiaotong University, Xi’an 710049, China; zhangchao007@stu.xjtu.edu.cn (C.Z.); wangxian@xjtu.edu.cn (X.W.); liyueming@xjtu.edu.cn (Y.L.); 2 Key Laboratory of Thermo-Fluid Science and Engineering, Ministry of Education, Xi’an Jiaotong University, Xi’an 710049, China; wentaoji@xjtu.edu.cn (W.J.); wqtao@xjtu.edu.cn (W.T.)

**Keywords:** silica aerogel, thermal conductivity, opacifier, pore size distribution

## Abstract

Due to their high-porosity, nanoporous structure and pores, aerogel materials possess extremely low thermal conductivity and have broad potential in the thermal insulation field. Silica aerogel materials are widely used because of their low thermal conductivity and high temperature resistance. Pure silica aerogel is very fragile and nearly transparent to the infrared spectrum within 3–8 μm. Doping fibers and opacifiers can overcome these drawbacks. In this paper, the influences of opacifier type and content on the thermal conductivity of silica fiber mat-aerogel composite are experimentally studied using the transient plane source method. The thermal insulation performances are compared from 100 to 750 °C at constant pressure in nitrogen atmosphere among pure fiber mat, fiber mat-aerogel, 20% SiC-fiber mat-aerogel, 30% ZrO_2_-fiber mat-aerogel and 20% SiC + 30% ZrO_2_-fiber mat-aerogel. Fiber mat-aerogel doped with 20% SiC has the lowest thermal conductivity, 0.0792 W/m·K at 750 °C, which proves that the proper type and moderate content of opacifier dominates the low thermal conductivity. The pore size distribution indicates that the volume fraction of the micropore and mesopore is also the key factor for reducing the thermal conductivity of porous materials.

## 1. Introduction

Aerogels, manufactured by sol-gel and different kinds of drying processes, are typical nanoporous materials [1]. Because of the high porosity, low density and nanoporous structure, aerogels possess outstanding features such as an extremely high specific surface area, low dielectric constant, and low thermal conductivity. Thus, aerogels have wide application prospects in the fields of adsorbtion, catalysts, thermal insulation, sensors, integrated circuits, etc. [2,3,4]. Aerogels are usually known as superinsulation materials because of their thermal conductivity, and can be much less than the thermal conductivity of still air [5]. Aerogels have the lowest thermal conductivity of solids; for example the thermal conductivity of silica aerogel can be as low as 0.013 W/m·K at room temperature and atmospheric pressure [6,7]. Thermal insulation materials have wide applications in energy saving in buildings, food cold chain and storage, liquefied gas transport, storage, boiler, furnace and pipe thermal insulation, aircraft thermal protection, spacesuits, etc. [5,8]. The global market of thermal insulation materials is estimated to be 50 billion US dollars in 2017 [9].

Heat transfer within porous material has three modes: heat conduction via solid skeletons and gas molecules, heat convection via gas molecules, and thermal radiation among solid skeletons. Heat convection in pores can be neglected with pore size is smaller than 1 mm [10]. The pore size of aerogels is mainly in the order of nanometers or dozens of nanometers, which is smaller than the mean free path of free gas molecules (70 nm for air at atmospheric pressure), thus the gaseous thermal conductivity within aerogels is much smaller than the thermal conductivity of free gas. The low gaseous thermal conductivity and large porosity make it possible that the thermal conductivity of aerogel can be smaller than that of still air [11,12]. In addition, due to the size effect of solid skeletons, the solid thermal conductivity is also smaller than that of bulk materials [7,13]. Many studies have been conducted to reveal the heat transfer mechanisms of aerogels [14], to measure, to predict and to optimize the thermal conductivity of aerogels and their composite [15,16,17,18,19,20].

Among different kinds of aerogels, silica aerogel materials are widely used for their low thermal conductivity and high temperature resistance. Pure silica aerogel is very fragile and nearly transparent to the infrared spectrum over the range of 3–8 μm, in which most thermal radiation is transported [21]. Therefore, reinforced fibers and opacifiers are doped with silica aerogel to enhance their mechanical properties and extinction ability to thermal radiation [16,18,21,22,23]. To obtain better thermal insulation property at high temperature, experimental characterization on the thermal conductivity and microstructure of the aerogel composite are conducted in the present study. To obtain the thermal conductivitiy of silica aerogel doped with fiber mat and ZrO_2_, SiC opacifiers of different contents are measured using the transient plane source method. The influences of the microscopic morphology and the pore size distribution on the thermal insulation property of the aerogel composite are also discussed. The materials studied are aerogel doped with fiber mat or pure fiber mat. When used in a thermal protection system or another thermal insulation field, these materials are placed in a non-bearing location. The bulk shape of these materials can be maintained during cutting, transporting and serving processes due to the relatively high mechanical strength of fiber mat. Thus, the mechanical properties of the materials doped with different additives is not investigated in this study.

## 2. Experimental Investigation

### 2.1. Test Materials

**Figure 1:**
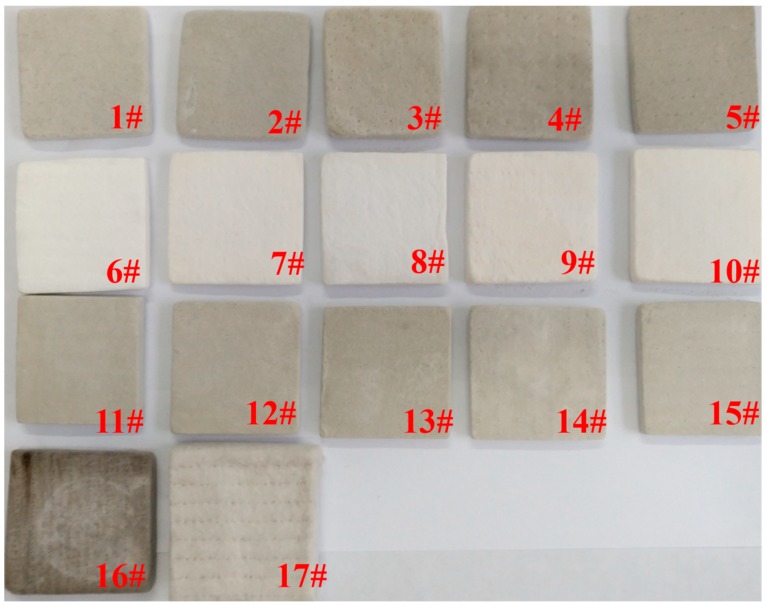
Test samples.

The silica fiber mat and silica aerogel doped with different opacifiers shown in Figure 1 are investigated. Their constituents, porosity and density are listed in Table 1. The silica aerogel is manufactured by sol-gel and supercritical processes. The present study mainly focuses on the thermal insulation performance of different opacifier-fiber-aerogel composites. The influences of components, temperature, and pore size distribution on the thermal conductivity are investigated. The studied materials are commercial products. For opacifier-fiber mat-aerogel composite, the fiber mat is impregnated with sols and the opacifiers are dispersed into the sols. Detailed synthesis processes can be referred to in references [24,25,26]. Figure 1 shows the image of 17 test samples. Sample 16 is darkened after heating at high temperature. Samples 1–5 are fiber mat-aerogel doped with different mass fractions of SiC opacifier while samples 6–10 are fiber mat-aerogel doped with different ZrO_2_ opacifiers. Both SiC (20% wt) and ZrO_2_ are doped in samples 11–15. Sample 16 is fiber mat synthesized with aerogel and sample 17 is pure fiber mat. The material density is determined by measuring the volume of material with regular shape and its weight after being dried at 150 °C in the hygrothermostat for more than 12 h. The densities of bulk ZrO_2_, SiC and SiO_2_ are 5.89 g/cm^3^, 3.22 g/cm^3^ and 2.1 g/cm^3^, respectively [18]. With the known mass fractions, densities of components and apparent material density, the volume fractions of components can be calculated. Then the porosity of the test sample can be determined. The porosity of these materials is within 87.5–94.8%.

### 2.2. Sample Characterization

The thermal insulation materials studied are constituted of silica aerogel matrix, silica fiber mat and opacifier. Multiscale components and multiscale pores exist in the composite. The diameter of fiber and opacifier is in the order of several microns while the aerogel matrix has pores with size from several nanometers to hundreds of nanometers. There are also micron cracks in the aerogel matrix. Micron-size pores exist in the fiber mat or its composite.

To characterize the multiscale pore size distribution and specific surface area, both the nitrogen adsorption method with ASAP2020 and mercury injection method with IV9500 are adopted. Nitrogen adsorption and desorption at 77 K is used to measure the micropore (<2 nm) and mesopore (2–50 nm) while mercury injection is used to measure the macropore (5 nm~1000 μm). A scanning electron microscope (FEI-Quanta400, FEI Company, Eindhoven, The Netherlands) and field emission scanning electron microscopes (JEOL-7800F, JEOL Ltd., Tokyo, Japan; Gemini SEM500, Carl Zeiss AG, Oberkochen, Germany) are used to observe the microscopic morphology.

### 2.3. Thermal Conductivity Measurement

The thermal conductivity of aerogel composite is measured with Hot Disk TPS2500S (Hot Disk AB, Gothenburg, Sweden), which is based on the transient plane source method. A double spiral sensor made of nickel is used both as a heater and a temperature monitor. The sensor is coated with Kapton or Mica insulation layer depending on the working temperature. The dynamic temperature of the sensor is recorded by measuring the electrical resistance of heat elements with known temperature coefficient of resistance. The test theory can be referred to in reference [27]. For low temperature measurement, the Kapton5501 sensor (Hot Disk AB, Gothenburg, Sweden) and test materials are placed in the temperature and humidity chamber, which provides controllable temperature and humidity in the atmosphere. For high temperature measurement, the Mica4922 sensor (Hot Disk AB, Gothenburg, Sweden) and test materials are placed in the tube furnace, which provides a high temperature environment.

Thermal conductivity is measured at 25 °C 15% RH by placing the test sample and sensor in the controllable temperature and humidity chamber. To avoid the influence of water vapor adsorbed by silica aerogel, the samples are dried at 120 °C for more than 12 h and then stored in sealed plastic bags. After the chamber reached the preset temperature and humidity, the samples were taken out and placed together with the sensor in the chamber. Thermal conductivity measurement is conducted when the temperature and humidity in the chamber is stable again. For each sample, the measurement is conducted at least three times for averaging purposes, and the interval time between the two adjacent tests is longer than one hour, to ensure the temperature within the sensor and test materials is uniform before the next transient test. In the atmosphere environment, the transient test is conducted using the Kapton5501 sensor (sensor radius ~6.403 mm, sensor thickness ~60 μm).

For thermal conductivity measurement at high temperature, a Mica-insulated sensor is adopted to resist the high temperature. As investigated in our previous work, the plane source assumption will be questionable when measuring extremely low thermal conductivity materials using the Mica sensor [28]. The thermal conductivity of thermal insulation material measured by Mica sensor is overestimated to account for a portion of energy dissipating from the sensor’s side. The uncertainty caused by heat dissipation via sensor radial direction is dependent on the sensor radius and thickness, thermal property of test material and Mica insulation layer. The uncertainty also varies with temperature, which is difficult to quantify. The heat dissipation from the Mica sensor side reduces greatly when using a larger-radius sensor. Thus, the Mica4922 sensor (sensor radius ~14.61 mm, sensor thickness ~220 μm) is used in the present study. However, the measured thermal conductivity is still overestimated. The Kapton sensor is thin and possesses high precision, but could only take measurements at temperatures lower than 150 °C. Thus, a modification factor is proposed by comparing the thermal conductivity measured by Kapton5501 sensor and Mica4922 sensor at 100 °C to reduce the uncertainty measured by Mica4922 sensor to some extent. A modification factor of 1.11 is obtained from the mean deviations of different materials and then the factor is used to modify the thermal conductivity measured by the Mica4922 sensor. The modified factor used in the study is a constant. The uncertainty of thermal conductivity measured by the Mica4922 sensor at high temperature is estimated to be no wider than ±5% after use of the modification factor. The uncertainty of thermal conductivity measured by the Kapton5501 sensor at room temperature is estimated to be ±3%.

The high temperature thermal conductivity is measured by the Mica sensor in the tube furnace. The furnace provides closed and controllable temperature environment. Before conducting the test, the air inside the tube system is replaced by high purity nitrogen. The tube furnace is pumped exhaustively to eliminate the absorbed water vapor and air in the test material and the tube furnace, then nitrogen is injected into the furnace. The tube furnace is pumped and filled with nitrogen at least three times to eliminate the air within the materials thoroughly. The valves on the tube furnace are closed when the pressure inside is about 100 kPa. The gas pressure inside the closed furnace will increase with temperature. When the tube furnace reaches the preset temperature, the gas pressure inside is adjusted with valves to constant pressure by utilizing the residual vacuum in the pipeline. The same procedure should be repeated at each temperature.

## 3. Results and Discussion

### 3.1. Thermal Conductivity

**Figure 2:**
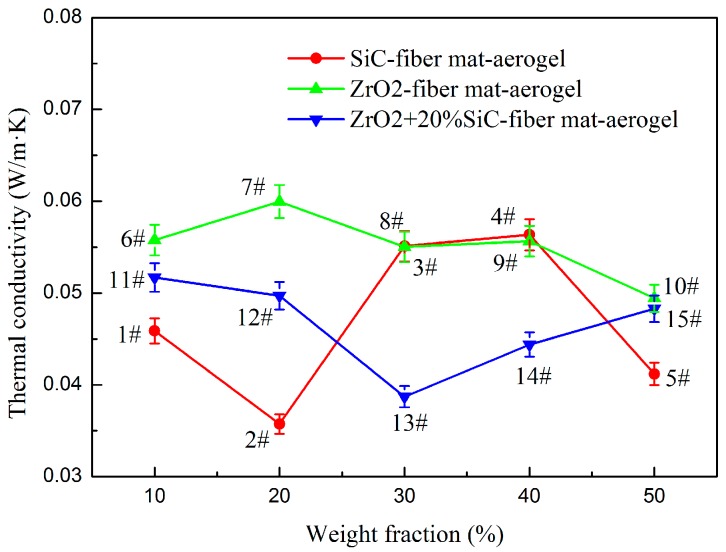
Thermal conductivity comparison with different opacifier content at 25 °C.

Figure 2 shows the thermal conductivity of samples 1–15 at 25 °C measured in the temperature and humidity chamber. For each sample, the thermal conductivity is measured by the Kapton5501 sensor at least three times. The figure shows the average value and the error bar (±3%). For SiC-doped fiber mat-aerogel, opacifiers with weight proportions of 10–50% are studied. Sample 2 has the lowest density (202.0 kg/m^3^) among the five fiber-loaded materials, and it possesses the lowest thermal conductivity. For ZrO_2_-doped fiber mat-aerogel, its thermal conductivity is also investigated for opacifier content ranging from 10–50%. Sample 8 (276.4 kg/m^3^) doped with 30% ZrO_2_ has better thermal insulation performance than sample 7 (255.4 kg/m^3^) and sample 9 (271.7 kg/m^3^), even though it has a higher density. Sample 10 (280.6 kg/m^3^) doped with 50% ZrO_2_ possesses the optimal thermal insulation among the five materials (10.2% less than that of sample 8) which implies that the reduction of thermal radiation is higher than the enhancement of solid heat conduction at this doping proportion and temperature. Attempts are also made by doping two different kinds of opacifiers simultaneously. Samples 11–15 (256.0–284.7 kg/m^3^) are 20% SiC-fiber mat-aerogel doped with different contents of ZrO_2_ particles. Sample 13 (263.8 kg/m^3^) doped with 30% ZrO_2_ and 20% SiC has best insulation performance among the five samples while its density is not the lowest one. The thermal conductivity of fiber mat-aerogel (Sample 16 with density of 262.9 kg/m^3^) is 0.05179 W/m·K at 25 °C while thermal conductivity of pure fiber mat (Sample 17 with density of 160.4 kg/m^3^) is 0.05073 W/m·K. Compared to samples 1–16, although sample 17 has the lowest density, it has a median thermal conductivity that proves the nanoporous aerogel and the opacifier are useful for improving thermal insulation even at room temperature.

The effective thermal conductivity of aerogel composite is influenced by the components, mass ratio, porosity, pore size distribution, diameter and orientation of fiber, type and size of opacifier, temperature, and gas pressure, etc. Both the solid heat conduction and thermal radiation increase rapidly with temperature. The doping of the opacifier will suppress the thermal radiation to some extent. Therefore, the minimum thermal conductivity at different temperatures cannot be obtained for the same concentration of opacifier. The optimal proportion of opacifiers varies with type, size, and temperature. The effective model can be used to predict and to optimize the thermal conductivity of aerogel composite. However, the reliability also relies on the effective model [18], the solid thermal conductivity of components at different temperature (which is difficult to determine accurately at nanoscale), and extinction coefficient. Therefore, the evaluation of thermal insulation performance should be conducted at various temperatures.

**Figure 3:**
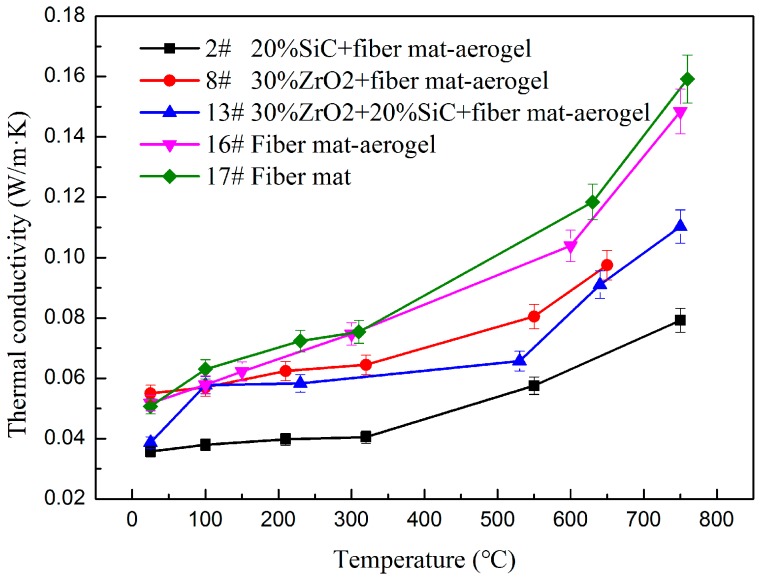
Thermal conductivity comparison at high temperature.

Opacifier doped in silica aerogels is used to restrain the thermal radiation. The adding of an opacifier inevitably enhances solid conductivity. The solid thermal conductivity of SiC is much larger than that of ZrO_2_ while the extinction coefficient of SiC is much higher than that of ZrO_2_ [18]. Since the overall effect of doping with an opacifier depends on the temperature, samples 2, 8, and 13 are selected and measured from 25 to 750 °C at constant pressure in nitrogen atmosphere and compared with fiber mat-aerogel (sample 16) and fiber mat (sample 17). It is worth noticing that although sample 10 has the lowest thermal conductivity among the five ZrO_2_-doped materials, sample 8 is selected for comparison at high temperature. The reason is that solid heat conduction will be enhanced greatly with the increment of temperature when doping with more ZrO_2_. The three samples are selected to study the effect of temperature. Comparison is also made with pure fiber mat and fiber mat-aerogel composite, and the results are shown in Figure 3. At each temperature, the sample is measured by Mica4922 sensor at least three times. The figure shows the average value and the error bar is ±5%. Sample 17 has the highest thermal conductivity at high temperature even though it possesses the lowest density (160.4 kg/m^3^). The thermal insulation of fiber mat can be improved after doping with silica aerogel. Thus, the thermal conductivity of sample 16 is lower than that of sample 17 even though its density is 64% higher (262.9 kg/m^3^). The thermal insulation performance of fiber mat-aerogel can be further enhanced by doping with opacifier. Although samples 8, 13, and 16 have similar density (276.4 kg/m^3^, 263.8 kg/m^3^, 262.9 kg/m^3^) the thermal conductivity of sample 13 is lower than that of the other two samples. Compared with sample 2 (202.0 kg/m^3^), the thermal conductivity of sample 13 increased more significantly at high temperature. It is because the enhancement of solid conduction by adding 30% ZrO_2_ is larger than the suppression of thermal radiation.

Compared to ZrO_2_, SiC has much higher solid thermal conductivity and extinction coefficient within 3–8 μm. Most of the thermal radiation will transfer via infrared spectrum with wavelength of 3–8 μm, which can easily be estimated using Wien’s displacement law. The larger the extinction coefficient, the smaller the radiative thermal conductivity. The combined effects of enhanced solid heat conduction and suppressed thermal radiation lead to the fact that the SiC opacifier is more efficient than ZrO_2_ opacifier. The results prove that the doping of opacifier could enhance the thermal insulation performance at high temperature and SiC opacifier has better performance due to its higher extinction coefficient.

According to our previous study of silica aerogel composite [18], the contribution of radiative conduction increases rapidly with temperature. The contributions of radiative conduction at 650 °C are 25.7% when doped with 44.2% ZrO_2_ (3.5 μm, 486.9 kg/m^3^), 9.3% when doped with 18.3% SiC (3.5 μm, 368.1 kg/m^3^) and 10.6% when doped with 30.2% SiC (3.5 μm, 387.1 kg/m^3^). For sample 2, 20% SiC-fiber mat-aerogel has density of 202.0 kg/m^3^ and thermal conductivity of 0.0792 W/m·K at 750 °C. In a previous study [18], SiC-fiber-aerogel with density of 387.1 kg/m^3^ and mass ratio of 30.2%:2.7%:67.1% has thermal conductivity of 0.0474 W/m·K at 700 °C. The latter one has lower thermal conductivity due to it having more SiC opacifier, although it has higher density. For sample 8, 30% ZrO_2_-fiber mat-aerogel has density of 276.4 kg/m^3^. It has thermal conductivity of 0.0975 W/m·K at 650 °C. In reference [18], ZrO_2_-fiber-aeorgel with density of 486.9 kg/m^3^ mass ratio of 53.6%:2.2%:44.2% has thermal conductivity of 0.0516 W/m·K at 650 °C. This proves that the aerogel composite possesses higher thermal insulation performance at high temperature if doping more opacifier. The doping of fiber mat with aerogel in the present work has higher mechanical strength compared to previous materials (aerogel powders are easily flaked off the sample in reference [18]).

### 3.2. Microscopic Morphology

**Figure 4:**
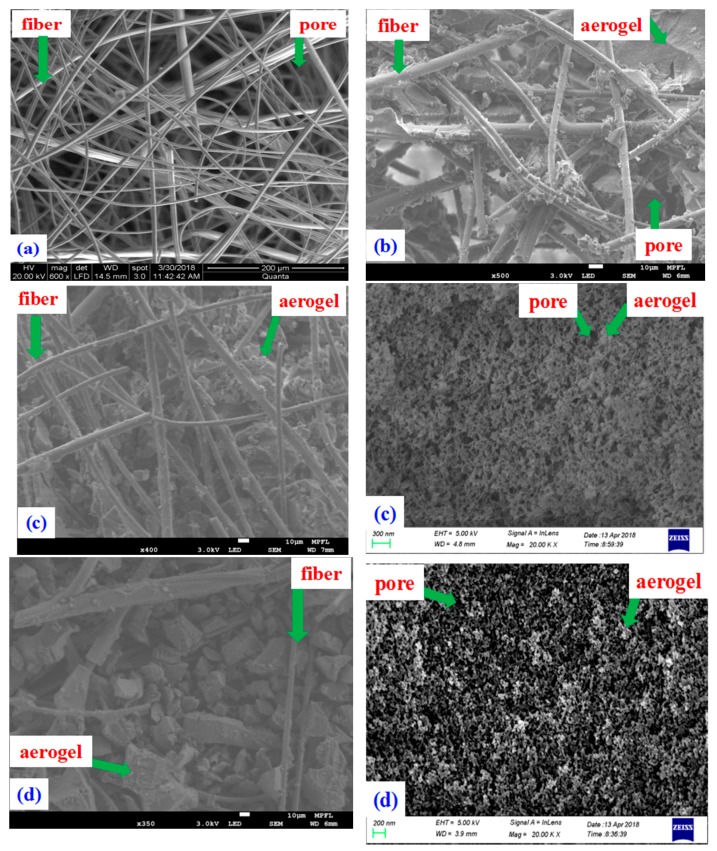
SEM of different porous materials. (**a**) Sample 17 Fiber mat (FEI-Quanta400); (**b**) Sample 16 Fiber mat-aerogel (JEOL-7800F); (**c**) Sample 8 30% ZrO_2_-fiber mat-aerogel (left: JEOL-7800F, right: GeminiSEM 500); (**d**) Sample 13 30% ZrO_2_ + 20% SiC-fiber mat-aerogel (left: JEOL-7800F, right: GeminiSEM 500).

To observe the microscopic morphology of these porous insulation materials, different microscopes are used. Due the composites having multiscale structures and being non-conductive, it is impossible to observe the multiscale microscopic morphology with one microscope. Thus, FEI-Quanta400 is used to scan the fiber mat (sample 17) and JEOL-7800F is used to measure the nanoporous structure of aerogel (sample 8 and sample 13). The rest of the SEM figures are determined by GeminiSEM 500 (Carl Zeiss AG, Jena, Germany). The fibers are distributed randomly in the plane perpendicular to the sample thickness while the porous structure can be regarded as homogeneous. Samples with a size of several millimeters is cut from bulk materials along the in-plane direction, and the cross-section area is observed. SEM pictures are obtained by the scanning microscopes, so only the sample surface topography is observed. Figure 4a is the SEM of pure fiber mat, in which the silica fibers are randomly distributed. Figure 4b shows the SEM of the fiber mat-aerogel composite. Micrcon-size pores are also observed in the composite. The micron pores are filled with silica aerogel. Figure 4c is the SEM of 30% ZrO_2_-fiber mat-aerogel. The SEM pictures of ZrO_2_-fiber mat-aerogel clearly show that the composite has multiscale pore size. Due to the existence of the fiber mat, there are plenty of pores with diameter of dozens of microns. For the aerogel matrix, there are plenty of pores with diameter in the order of hundreds of nanometers. The SEM results can be used to confirm the pore size distribution determined by the mercury injection method. Sample 13 doped more opacifier (20% SiC) than sample 8 and its SEM is shown in Figure 4d. The diameter of the fibers can be easily extracted from the SEM pictures. The mean diameter of fibers is ~6 μm and the error is estimated within ±1.5 μm. Due to the composites having pores with size from several nanometers to dozens of microns, and the SEM pictures being unable to exhibit all the pores, it is difficult to give a mean value of the pore size. The pore size of the composites can be observed from the pore size distribution measured from nitrogen adsorption and mercury injection tests.

### 3.3. Specific Surface Area and Pore Size Distribution

**Figure 5:**
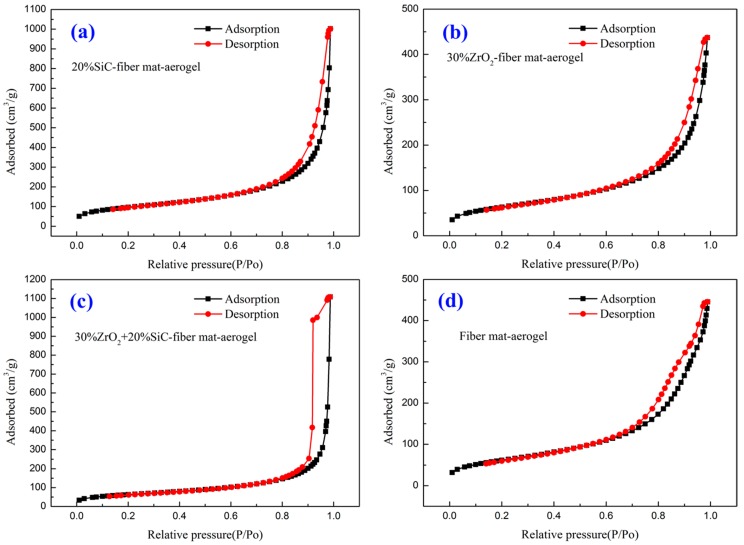
Adsorption and desorption isotherms. (**a**) Sample 2# 20% SiC-fiber mat-aerogel; (**b**) Sample 8# 30% ZrO_2_-fiber mat-aerogel; (**c**) Sample 13# 30% ZrO_2_ + 20% SiC-fiber mat; (**d**) Sample 16# Fiber mat-aerogel.

The SEM pictures show the microscopic morphology visually. To obtain the microscopic structure property quantitatively, the specific surface area and pore size distribution of the above porous materials are measured by nitrogen adsorption method and mercury injection method. The materials studied have multiscale pores. The combined nitrogen adsorption method and mercury injection method provides a choice of characterizing the pore size distribution from several nanometers to dozens of microns. Figure 5 is the adsorption and desorption isotherms of four aerogel composites. Adsorption and desorption hysteresis is found in all four materials due to the existence of nanopores. The specific surface areas measured from the adsorption of nitrogen and mercury are listed in Table 2.

**Figure 6:**
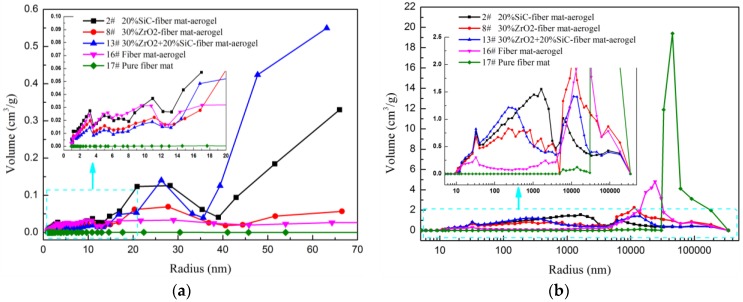
Pore size distribution of different materials. (**a**) Measured by Nitrogen adsorption; (**b**) Measured by Mercury injection.

Sample 2 has the largest specific surface area among the five materials, which implies that sample 2 has a large volume of micropore and mesopore, which can be proved in the pore size distribution, as shown in the inset of Figure 6a. The specific surface area determined by nitrogen adsorption method is much larger than the mercury injection method due to the nitrogen adsorption method being suitable for micropore and mesopore while the mercury method would destroy these kinds of pores. Smaller pores have larger specific surface area than larger pores at constant pore volume. The specific surface area of pure fiber mat is negligible when compared to the aerogel composite. It also implies that only the macropore exists in the fiber mat, which can be verified by pore size distribution as shown in Figure 6b. As shown in Figure 6b, all five materials have micron-size pores. Compared with pure fiber mat, the volume fraction and size of micron pores decreased after doped with aerogel. The volume of nanopores increased for fiber mat doped with aerogel. Sample 2 has more pores smaller than 20 nm compared with other materials, which plays an important role in reducing thermal conductivity, since the gas motion within such small pores is suppressed [12,29,30].

The aerogel matrix has plenty of pores with diameter in the range of several nanometers to dozens of nanometers while the fiber mat has large volume of micron-size pores. When synthetizing aerogel with fiber mat, aerogel will fill the pores of the fiber mat to some extent. Due to the existence of cracks in aerogel and non-ideal filling of aerogel, the fiber-aerogel composite has a multiscale porous structure, and ranges from several nanometers to hundreds of microns. Although the mercury injection method would destroy the structure of aerogel materials, the nitrogen adsorption method can only measure nano-pores with size from 2 to 50 nm. Thus, the mercury technique is adopted to measure macropore with size from 5 nm to hundreds of microns. Although this characterization method cannot measure pore size exactly, the pore size distribution can be obtained quantitatively. In addition, the pore size distributions obtained by both methods has similar variation in the overlap size range. For example, the pore size distributions of samples 2, 8, and 13 determined from nitrogen adsorption and mercury injection has similarity within the pore size ranges from 10 to 60 nm, which can be observed from the insets of Figure 6. However, due to the differences of test theory and absorbent molecules, the absolute values of pore volume determined by two methods are not identical. Regardless, the pore size distribution can also be verified by SEM images. The nitrogen adsorption method and mercury injection method have also been widely used to evaluate the multiscale pore size distribution of aerogel materials [31,32,33,34].

## 4. Conclusions

The thermal conductivity and microscopic structure property of different kinds aerogel composite were investigated experimentally. The thermal conductivity of silica fiber mat-aerogel doped with different contents of SiC, ZrO_2_, SiC + ZrO_2_ opacifier were measured at 25 °C. Fiber mat-aerogel doped with 20% SiC particles has thermal conductivity as low as 0.0357 W/m·K. To evaluate the shading effect of opacifier at high temperature, the thermal conductivity of fiber mat, fiber mat-aerogel, fiber mat-aerogel doped with 20% SiC, 30% ZrO_2_ and 20% SiC + 30% ZrO_2_ are measured at temperatures as high as 750 °C at constant pressure in a nitrogen atmosphere. The results prove that the adding of an opacifier enhances the thermal insulation performance of a fiber mat-aerogel composite. Fiber mat-aerogel doped with 20% SiC has the lowest thermal conductivity, 0.0792 W/m·K at 750 °C, which is only half the value of pure fiber mat. The thermal conductivity of fiber mat-aerogel doped with 20% SiC at high temperature is also lower than that doped with 20% SiC + 30% ZrO_2_ even though they have little difference at 25 °C. Doping more opacifier will suppress more thermal radiation but will transfer more heat via conduction. This finding proves that there exists an optimal doping content of opacifier, and the shading effect of SiC is better than that of ZrO_2_.

The SEM pictures of insulation materials show the microscopic morphology of the fiber aerogel matrix visually. The diameter of fiber is about 6 μm. The pore size within fiber mat is in the order of dozens of microns. When doping with aerogel, the size and amount of micron pores decrease greatly. Meanwhile, the amount of micropore and mesopore increases and contributes more to the reduction of thermal conductivity, since the gas heat conduction within the pores is suppressed.

## Figures and Tables

**Table 1:** Test materials.

Sample	Material	Density (kg/m^3^)	Porosity
1	10% SiC-fiber mat-aerogel	244.7	89.7
2	20% SiC-fiber mat-aerogel	202.0	92.6
3	30% SiC-fiber mat-aerogel	267.6	91.8
4	40% SiC-fiber mat-aerogel	290.8	92.7
5	50% SiC-fiber mat-aerogel	265.8	94.8
6	10% ZrO_2_-fiber mat-aerogel	242.5	89.2
7	20% ZrO_2_-fiber mat-aerogel	255.4	89.4
8	30% ZrO_2_-fiber mat-aerogel	276.4	89.4
9	40% ZrO_2_-fiber mat-aerogel	271.7	90.4
10	50% ZrO_2_-fiber mat-aerogel	280.6	90.9
11	10% ZrO_2_ +20% SiC-fiber mat-aerogel	277.6	88.6
12	20% ZrO_2_ + 20% SiC-fiber mat-aerogel	255.4	89.6
13	30% ZrO_2_ + 20% SiC-fiber mat-aerogel	263.8	90.4
14	40% ZrO_2_ + 20% SiC-fiber mat-aerogel	256.0	91.4
15	50% ZrO_2_ + 20% SiC-fiber mat-aerogel	284.7	91.9
16	Fiber mat-aerogel	262.9	87.5
17	Pure fiber mat	160.4	92.4

**Table 2:** Specific surface area of aerogel composite.

Sample	N_2_ Adsorption-BET (m^2^/g)	N_2_ Adsorption-Langmuir (m^2^/g)	Hg Injection (m^2^/g)
2	343.5	508.6	71.4
8	222.7	328.7	51.7
13	224.8	333.1	64.0
16	222.0	329.1	71.4
17	1.5	2.3	0.44
